# Effect of Steam and Smoke Cooking Processes on Web-Foot Octopus (*Amphioctopus* sp.) Home Meal Replacement Product

**DOI:** 10.3390/foods10112825

**Published:** 2021-11-16

**Authors:** Bertoka Fajar Surya Perwira Negara, Hee-Jin Gong, Mi-Jeong Lee, Jae-Suk Choi

**Affiliations:** 1 Seafood Research Center, IACF, Silla University, 606, Advanced Seafood Processing Complex, Wonyang-ro, Amnam-dong, Seo-gu, Busan 49277, Korea; ftrnd12@silla.ac.kr (B.F.S.P.N.); ftrnd2@silla.ac.kr (H.-J.G.); ftrnd10@silla.ac.kr (M.-J.L.); 2 Department of Food Biotechnology, College of Medical and Life Sciences, Silla University, 140, Baegyang-daero 700 beon-gil, Sasang-gu, Busan 46958, Korea; 3 Department of Marine Science, University of Bengkulu, Jl. WR. Soepratman, Bengkulu 38371, Indonesia

**Keywords:** web-foot octopus, steam, smoke, HMR, nutritional

## Abstract

In Korea, the web-foot octopus (*Amphioctopus* sp.) is commonly consumed *as jjukkumi bokkeum*, a spicy stir-fried octopus dish. Using steaming and smoking methods, we made *jjukkumi bokkeum* home meal replacement (HMR) products. The response surface methodology (RSM) was employed to optimize the steam and smoke processes. Quick freezing was applied to freeze the test product at −35 °C. Then, the physicochemical, biological, nutritional characteristics, and shelf-life of the test HMR products were evaluated. The optimal conditions for steaming and smoking were 95 °C for 2 min and 70 °C for 11 min, respectively. The pH, volatile basic nitrogen content, and thiobarbituric acid-reactive substances content decreased after steaming and smoking, indicating that these processes maintained these parameters well. Sensory evaluation revealed that there were no changes in these characteristics after freezing and reheating. Further, the test HMR products contained the daily nutritional requirements of macro and micronutrients, as well as amino acids and fatty acids. The shelf-life of the HMR products was estimated to be 15 months. The findings of this study indicate that the application of steam and smoke processes to produce a *jjukkumi bokkeum* HMR product results in a high-quality product with a long shelf-life.

## 1. Introduction

In Korea, the demand for home meal replacement (HMR) products is rapidly growing. According to the type, HMR products can be divided into four categories: Ready to eat, ready to cook, ready to prepare, and ready to heat [1]. Seafood HMRs are one of the most popular products and have high demand because of their nutritional value, ease of preparation, and long shelf-life. Several studies have been conducted to develop seafood-based HMRs, including mackerel [2,3,4], a mixture of pen shell adductor muscles and common squid meat [5], and yellowfin sole (*Pleuronectes aspera*) [6]. While several types of seafood, such as the web-foot octopus, have the potential to become successful HMRs, seafood-based HMR products are still under development and have not yet been introduced to the market.

The web-foot octopus (*Amphioctopus* sp.) is a member of the molluscan class Cephalopoda and is commonly found in the Pacific Ocean. As a low-calorie and low-fat seafood, octopus contains high levels of nutrients, including omega-3 fatty acids, protein, vitamin B-12, potassium, magnesium, calcium, taurine, and eicosapentaenoic acid (EPA) [7]. These nutrients have beneficial effects on human health, including lowering blood pressure, reducing the risk of stroke, reducing lipid hydroperoxide formation in the body, and maintaining good heart health [8].

In Korea, the web-foot octopus is commonly consumed as the main ingredient in *jjukkumi bokkeum*, which is a popular food item. Owing to the popularity of *jjukkumi bokkeum*, there is an untapped market potential to develop the dish as an HMR product. However, meeting this potential demand requires a comprehensive understanding of the cooking methods that can be used to maintain the texture, nutritional, and taste characteristics, and thereby ensure a high-quality HMR *jjukkumi bokkeum* product.

Several studies have demonstrated that the combination of steaming and smoking seafood is highly efficient and has many advantages. The application of steam to process seafood has several advantages such as good texture and food appearance [9], reduced food oxidation [10], improved product quality, and shortened cooking time [11]. Meanwhile, the use of smoke to prepare seafood can increase sensory parameters, such as aroma, taste, color, and texture [12], and reduce microbial growth [13], while maintaining the nutritional value of the smoked product [14].

To ensure that consumers receive the best quality, including texture, nutrition, and taste, *jjukkumi bokkeum*, both steam and smoke cooking methods should be employed. Therefore, we developed a novel HMR *jjukkumi bokkeum* product, which consists of a web-foot octopus mixed with Korean sauce. We also evaluated the application of steam and smoke methods to produce high-quality HMR *jjukkumi bokkeum*. The findings of this study can be adopted in the food industry.

## 2. Materials and Methods

### 2.1. Preparation of Raw Materials

Frozen web-foot octopus was provided by Haesong Fish and Good Co., Ltd. (Busan, Korea). The size of frozen web-foot octopus used in this study was approximately 60 g/pcs. The frozen web-foot octopus was cleaned and double cut belly. We used 100 pcs of frozen web-foot octopus (approximately 6 kg) as raw material, followed by thawing under three conditions: Water thawing (WT), air thawing (AT), and high-frequency defrosting (HFD). The filter paper wetness method was used to measure the drip loss [15] of the frozen web-foot octopus by weighing the No. 2 filter paper (55 mm; Advantech, Tokyo, Japan) before being placed on the sample (y). Then, the frozen web-foot octopus was placed in a plastic bag for WT and AT. Samples undergoing the WT method were placed under flowing tap water (22–24 °C), while the AT samples were thawed at ambient temperature (20–25 °C). A TEMPERTRON FRT-10 HFD machine (Yamamoto Vinita Co. Ltd., Osaka, Japan) was used for the HFD method, in which the power was set at 27 MHz for 10 min. After the thawing processes were completed, the filter paper containing the exudates was weighed (x). The exudate value was calculated as the weight difference before and after thawing. Then, the drip loss (%) was determined using the following formula:

(1) $$\mathrm{Drip}\;\mathrm{loss}\;\left(\%\right)=\frac{x-y}{x}\times 100$$

### 2.2. Steaming and Smoking Processes

**Figure 1:**
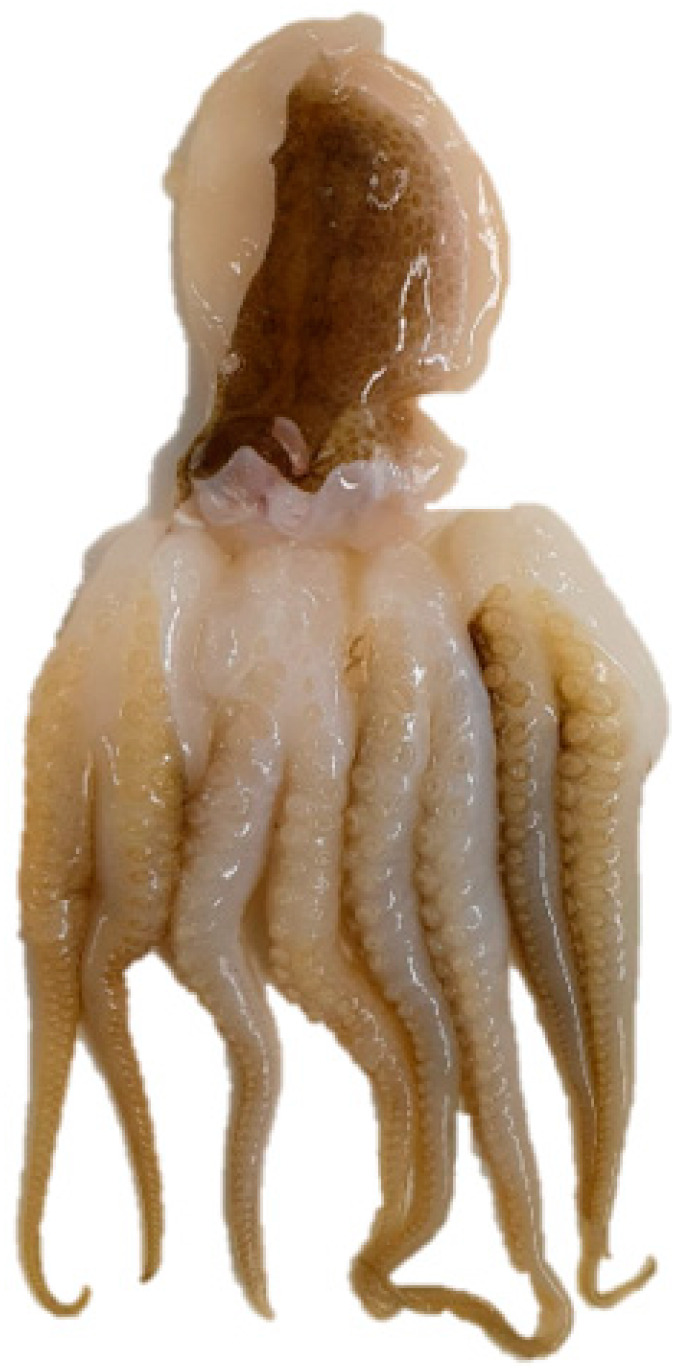
Web-foot octopus (*Amphioctopus* sp.) used in this study as a raw material for processing a new home meal replacement (HMR) product.

The thawed web-foot octopus (Figure 1) were cleaned, washed, and cut into 4-cm pieces. The samples were cooked using two methods: Steaming and smoking. Steaming was performed prior to smoking.

A steamer machine (CHDC–500, Chamco Chungha Co., Ltd., Busan, Korea) was used to steam the samples. The temperatures were set to 88 °C for 2 min, 90 °C for 1 min and 3 min, 95 °C for 0.6 min, 2 min, and 3.4 min, 100 °C for 1 min and 3 min, and 102 °C for 2 min. These temperatures and steaming times are based on the uncoded value of the response surface methodology (RSM).

The steamed octopus was smoked using a smoking machine (BDSTD6, BRAAI, Delta, BC, Canada). An oak sawdust puck was used to generate smoke. Similar to the steam process, the temperature and smoking duration were determined based on the uncoded value of the RSM. The temperatures and durations were: 56 °C for 10 min, 60 °C for 5 min and 15 min, 70 °C for 2.9 min, 10 min, and 17.1 min, 80 °C for 5 min and 15 min, and 84 °C for 10 min.

### 2.3. Preparing Test HMR Products

**Figure 2:**
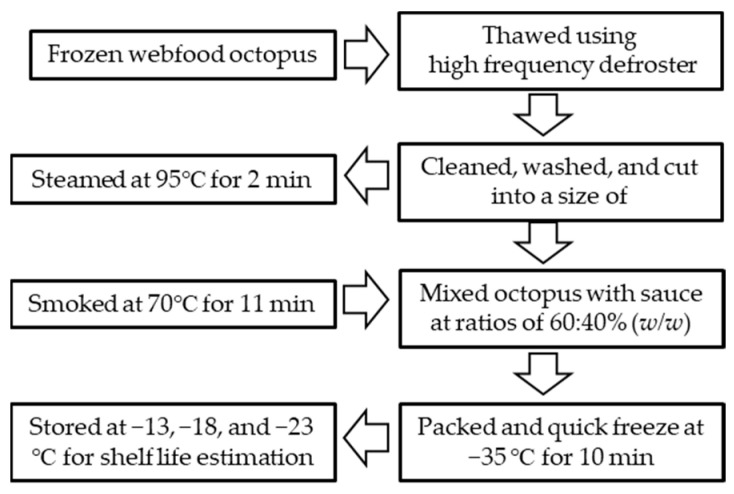
Schematic of processing web-foot octopus (*Amphioctopus* sp.) as a home meal replacement (HMR) product.

The *jjukkumi bokkeum* sauce was prepared before it was added to the cooked octopus. The sauce ingredients are listed in Table 1. The HMR products were produced by mixing web-foot octopus with sauce at a ratio (*w*/*w*) of 60:40. A polypropylene plastic bowl (New Ecopack Co. Ltd., Jeonju, Korea) was used to package the sample products, and was then sealed with a plastic film (K-Pack Co., Ltd., Hwanggan, Korea) using a tray sealing machine (TPS-TS3T, TPS Co. Ltd., Hwasung, Korea) at 180 °C for 5 s. A quick freezer (QF-700, Alpha Tech Co. Ltd., Incheon, Korea) was used to freeze the sample products at −35 °C for 10 min. The frozen products were stored at −13 °C, −18 °C, and −23 °C in a deep freezer (DF35035, IlShin BioBase Co. Ltd., Dongducheon, Korea) for shelf-life testing (Figure 2).

### 2.4. Sensory Evaluation

The aroma, color, flavor, texture, and overall acceptance were evaluated as sensory characteristics. An RE-M50 microwave (Samsung Electronics Co. Ltd., Seoul, Korea) was used to reheat the test HMR products for 90 s (700 W). In this analysis, 21 trained and certified sensory evaluation panelists were employed according to the Silla University Institutional Review Board (Approval No. 1041449-202104-HR-006; 23 April 2021). A hedonic scale of 1 to 9 was used to evaluate the sensory characteristics of the test HMR products (Table 2). Score 1 indicated “remarkably dislike” and 9 indicated “extreme like”. Five was considered the threshold value; any sample less than a score of 5 was considered unacceptable [16].

### 2.5. Physicochemical Analysis

The effects of steam and smoke processes on the physicochemical properties of the web-foot octopus were analyzed and compared with those of the raw web-foot octopus. These physicochemical properties included pH, VBN, and TBARS.

#### 2.5.1. pH

The sample was mixed with triple-distilled water at a ratio of 1:9 (*w*/*v*) using a homogenizer (SHG-15D, SciLab Co. Ltd., Seoul, Korea). The pH of the homogenized samples was measured using a pH meter (ST 3100, Ohaus Co., Parsippany, NJ, USA).

#### 2.5.2. Volatile Basic Nitrogen

The volatile basic nitrogen (VBN) contents were determined by homogenizing 5 g of a sample with 25 mL of triple-distilled water in a homogenizer (WiseTis SHG-15D, SciLab Co. Ltd., Seoul, Korea). The supernatant was separated from the homogenized material via centrifugation and filtration. Conway microdiffusion was used to analyze the supernatant. Conway cells were incubated for 90 min at 37 °C and titrated with 0.01 N NaOH [13].

#### 2.5.3. Thiobarbituric Acid-Reactive Substances

The thiobarbituric acid-reactive substances (TBARS) content was analyzed following the method described in Peiretti et al. [17]. Briefly, 5 g of each sample and 12.5 mL of trichloroacetic acid (20%) were homogenized in phosphoric acid (2 M) and adjusted to a total volume of 25 mL with distilled water. Centrifugation was performed to separate the supernatant at 1500 rpm for 10 min. A thiobarbituric acid solution (5 mM) was added to the supernatant at a ratio of 1:1 (*v*/*v*) and incubated for 30 min at 95 °C. A Spectrostar Nano (BMG Labtech Ltd., Ortenberg, Germany) was used to measure the absorbance of the sample at 530 nm.

### 2.6. Nutritional Quality Analysis

The nutritional contents of the test HMR product were analyzed following the Association of Official Analytical Chemists method [18]. GCMS-QP2020 gas chromatography mass spectrometer (Shimadzu Co., Kyoto, Japan) and 30 m × 0.25 mm DB-wax capillary column (Agilent Technology, Santa Clara, CA, USA) were used to analyze the fatty acid methyl esters by using EN 14078 standard mixture (Paragon Scientific Ltd., Wirral, UK) to identify the peaks. Moreover, amino acid analyzer (L-8900, Hitachi High-Tech Corp., Tokyo, Japan) was used to assess amino acid profiles. Fatty acid and amino acid profiles of the test HMR product were obtained according to a previous study [5].

### 2.7. Microbial Analysis

The total bacterial count (TBC), *Salmonella* spp., *Escherichia coli*, and *Staphylococcus aureus* colonies were measured as described by Negara et al. [5]. The sample was homogenized in sterile bags with sterile saline at a ratio of 1:9 (*w*/*v*) using a Stomacher 400 Circulator (Seward Ltd., West Sussex, UK) for 3 min. Difco plate count agar (BD Co., Franklin Lakes, NJ, USA), EC medium (BD Co., Franklin Lakes, NJ, USA), and Sanita-Kun plates (JNC Corp., Tokyo, Japan) were used to measure the TBCs, coliforms, *Salmonella* spp., and *Staphylococcus* spp., respectively. The plates were incubated at 35 °C for two days in an incubator (SIR-20, SciLab Co. Ltd., Seoul, Korea).

### 2.8. Shelf-Life Analysis

The product shelf-life was estimated following the guidelines of the Ministry of Food and Drug Safety, Republic of Korea. A program simulation (https://www.foodsafetykorea.go.kr, accessed on 26 August 2021) was used to estimate the shelf-life of the product by inputting the sensory evaluation score and the number of microorganisms (*E. coli, Staphylococcus aureus*, and *Salmonella* spp.). The shelf-life estimation was conducted for 90 days, and the samples were stored at −13 °C, −18 °C, and −23 °C.

### 2.9. Statistical Analysis

All experiments were performed in triplicate (*n* = 3), and the data are presented as the mean standard deviation (SD). Drip loss, TBC, and sensory properties were analyzed via a one-way analysis of variance at a 95% level of probability (*p* < 0.05) with IBM SPSS (version 23.0; IBM Corp., Armonk, NY, USA). The RSM was analyzed using Minitab v. 14.0 (Minitab Inc., Birmingham, UK). Temperature and cooking duration were set as the independent variables, while overall acceptance was set as the dependent variable.

## 3. Results and Discussion

### 3.1. Drip Loss

**Figure 3:**
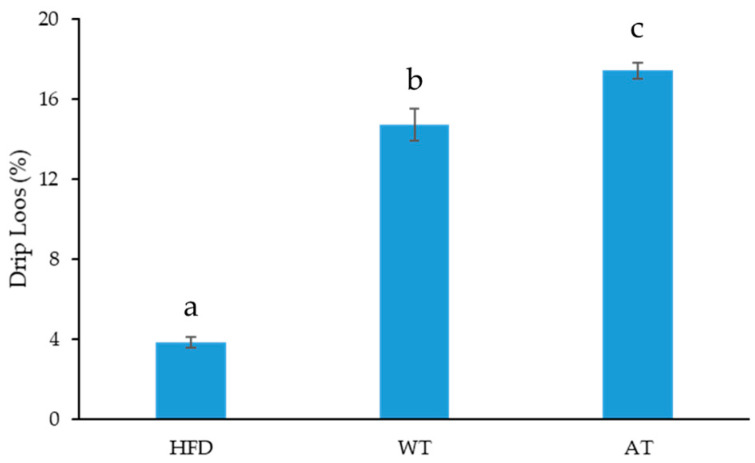
Drip loss analysis of web-foot octopus (*Amphioctopus* sp.) under different thawing methods. Data are presented as mean ± standard deviation. Different superscript letters (a–c) denote significantly different values according to Duncan’s test (*p* < 0.05).

Before assessing the effects of steam and smoke cooking processes, we measured the drip loss. As shown in Figure 3, we evaluated three different thawing methods. The results showed that the thawing method employed affected the drip losses, which ranged from 3.84% to 17.42%. Specifically, HFD resulted in the lowest drip loss, followed by WT, and AT. The drip loss after HFD was significantly lower (*p* < 0.05) than that of the other methods. This result is consistent with the results reported by Tirtawijaya et al. [2], Negara et al. [4], and Negara et al. [5], who found that thawing via HFD resulted in a lower drip loss, as compared with conventional thawing methods.

The duration of thawing affected the drip losses. In this study, the duration of thawing in the HFD was shorter than that in the WT and AT. To achieve a defrosted state, the HFD method only required 10 min, while WT and AT required 70 min and 90 min, respectively. A shorter thawing duration successfully minimizes damage to cell membranes and reduces drip loss [19,20]. Moreover, rapid thawing can decrease the mechanical damage caused by recrystallization [20]. The change in water position in the muscle of the web-foot octopus could also be prevented by rapid thawing, thereby maintaining the texture and nutritional value of the product.

According to [21], the thawing process for frozen fish and fishery products should be performed as quickly as possible. Water shifting from its original position during the thawing process leads to drip loss, resulting in a dry, stringy, and less tasty fish. Nutrient losses, such as proteins, vitamins, and minerals, can occur with drip loss; high drip loss is often linked to protein denaturation. In addition, high drip loss decreases attractiveness, nutritional value, texture, and appearance [22]. Moreover, Venugopal [19] and Alizadeh et al. [20] mentioned that a short thawing duration maintains quality and minimizes mechanical damage to cell membranes of products. The water-soluble proteins leach out during thawing periods, which could also lower the quality of the product.

A high drip loss decreases the sensory qualities and nutritional contents of thawed products [21,22]. In particular, some nutrients, such as proteins, minerals, and vitamins, leach during thawing [22]. HFD achieved the lowest drip loss, indicating that the use of HFD for thawing frozen web-foot octopus can effectively maintain the quality of thawed web-foot octopus. Therefore, HFD was selected for use in the development of the proposed HMR product.

### 3.2. Optimal Conditions for Steaming and Smoking

In this study, both steam and smoke were used to process the HMR products. We optimized the cooking conditions using the RSM for each method, in which the temperature (X_1_) and duration (X_2_) were set as independent variables, and overall acceptance (Y) was set as the dependent variable. As processing the web-foot octopus can decrease its sensory parameters, we selected the overall acceptance as the dependent variable to reflect the effect of different temperatures and duration on the steaming and smoking processes. To determine the optimal cooking temperature and duration, we used a five-level central composite design that generated 11 runs, which consisted of low, central, and high factor levels. The effect of two factors (Y) was modeled using a polynomial response surface with the prediction values using the following equation:

Y = b_0_ + b_1_X_1_ + b_2_X_2_ + b_11_X_1_^2^ + b_22_X_2_^2^ + b_12_X_1_X_2_

where b_0_, b_1_, b_2_, b_11_, b_22_, and b_12_ are the constant regression coefficients of the model, and X_1_ and X_2_ are the independent variables. The result of this composite design was the optimal steaming and smoking conditions.

**Figure 4:**
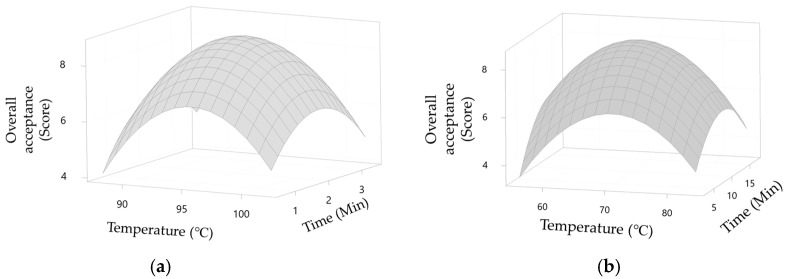
Three-dimensional response surface plots of web-foot octopus (*Amphioctopus* sp.) during (**a**) steaming and (**b**) smoking processes using RSM.

The model equations for steaming and smoking web-foot octopus are listed in Table 3. The *R*^2^ values of these models were 98% and 97% for steaming and smoking, respectively, indicating that the models were significant at a 95% confidence level (*p* < 0.05) and thus sufficient for estimating the best temperature and duration. During the steaming process, increasing the temperature and duration increased the overall acceptance score until a peak was reached (Figure 4a). The overall acceptance of web-foot octopus decreased at over 95 °C for 2 min. Under these conditions, the web-foot octopus became overcooked and had a lower chewiness. Lower temperatures and shorter steaming durations resulted in uncooked web-foot octopus. A similar pattern was observed for the smoking process. In particular, the overall acceptance score increased as the temperature and duration increased until optimal conditions were reached (Figure 4b) at 70 °C and 11 min.

**Figure 5:**
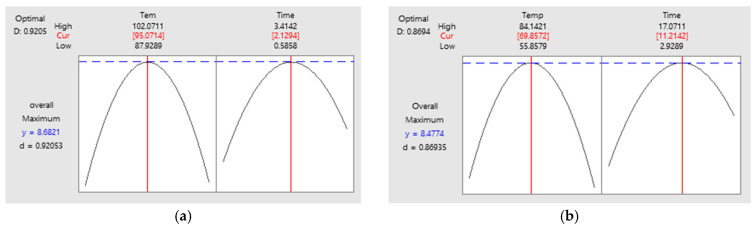
Response optimization of web-foot octopus (*Amphioctopus* sp.) in steam (**a**) and smoke (**b**) processes using response surface methodology (RSM).

Therefore, the optimal conditions for steaming and smoking were 95 °C for 2 min and 70 °C and 11 min, respectively. Under these conditions, the best overall acceptance score was obtained. Steaming the web-foot octopus at 95 °C for 2 min resulted in an overall acceptance score of 8.68 (Figure 5a), while smoking the web-foot octopus at 70 °C for 11 min resulted in an overall acceptance score of 8.47 (Figure 5b). Using the best combination of independent and dependent factors in the RSM, the optimal conditions were determined [23,24]. These conditions were used to steam and smoke the web-foot octopus to produce the test HMR product and were analyzed in subsequent experiments.

### 3.3. Effects of Steam and Smoke on Physicochemical Properties of Web-Foot Octopus

The effects of the steam and smoke processes on the physicochemical properties of the web-foot octopus were measured and compared with those of the raw web-foot octopus. Specifically, the physicochemical properties, which included pH, VBN, and TBARS, were evaluated to determine the freshness and nutrient oxidation of the web-foot octopus during these processes. The physicochemical properties of raw and steamed-smoked web-foot octopus are summarized in Table 4.

Overall, the steam and smoke processes increased the pH by approximately 1.8%, as compared with the raw web-foot octopus. These results are consistent with those of Sutikno et al. [25], Mohibbullah et al. [26], and Negara et al. [5], who found that cooking processes increased product pH. However, in this study, the increase in pH was not significant (approximately 1.8%), indicating the steam and smoke processes can maintain the freshness of the web-foot octopus. According to Yildiz [27], the pH of fresh seafood is neutral. Moreover, the decomposition of nitrogenous compounds in a web-foot octopus can be prevented by increasing the pH during these processes.

The other parameter that can determine the quality of seafood products is the VBN content. Herein, the steam and smoke processes decreased the VBN content by approximately 41.2%, as compared with the raw web-foot octopus. The results of the VBN content indicate that these processes can effectively reduce the spoilage process rate. According to Yildiz [27], an increase in VBN content indicates the activity of proteolytic bacteria and endogenous enzymes that yield nitrogenous materials. Furthermore, steam and smoke can prevent the degradation of proteins and amines in the web-foot octopus. The formation of trimethylamine-N-oxide, trimethylamine, dimethylamine, and formaldehyde, and the deamination of adenine nucleotides, which increase the VBN content, can also be avoided during steam and smoke processes. Chen et al. [28], Howgate [29], and Servillo et al. [30] found that the degradation of proteins and amines resulted in the formation of trimethylamine-N-oxide, trimethylamine, dimethylamine, and formaldehyde, thereby affecting the VBN content.

The TBARS level in seafood products can determine the rate of spoilage. In this study, after smoking and steaming, the TBARS value decreased by approximately 0.06 mg MDA/kg, as compared to that of raw web-foot octopus. This indicates that lipid oxidation is maintained during steam and smoke processes. Furthermore, according to the TBARS results, web-foot octopus processed using steam and smoke can be categorized as a perfect product. Yildiz [27] categorized seafood products based on the value of TBARS into perfect products (<3 mg MDA/kg), good products (5–5 mg MDA/kg), and consumable limit (7–8 mg MDA/kg).

The physicochemical property results indicate that steam and smoke processes maintain the integrity of the product well. In addition, these processes can preserve the freshness of the cooked web-foot octopus and prevent the oxidation of proteins and lipids. These conditions slow the spoilage rate. Moreover, the quality of the cooked web-foot octopus can be classified as a perfect product, thereby affecting the HMR product quality.

### 3.4. Sensory Evaluation of test HMR Product

The sensory characteristics of the test HMR product were tested to assess the quality of the product both before and after freezing. In this study, we used two different freeze methods: Slow and quick freezing. Prior to the sensory evaluations, the frozen test HMR products were reheated. The results showed that aroma, color, flavor, and overall acceptance did not significantly differ between the fresh and frozen test HMR products (Table 5). However, with respect to texture characteristics, slow freezing produced significantly different results, as compared with the fresh product, whereas quick freezing produced similar results. Slow freezing decreased the texture characteristics by approximately 4.7% compared to the fresh product. Similar results were reported by Tirtawijaya et al. [2], who found that the application of slow freezing decreased the texture of braised mackerel by 5.67%, as compared with fresh products.

These results indicate that the freezing method applied impacts the sensory characteristics of frozen products. During slow freezing, the growth of microorganisms and enzymatic activity are inhibited, but ice crystal formation affects membrane disruption, leading to a decrease in texture and oxidation [31]. According to Hergenreder et al. [32], slow freezing produces large extracellular ice crystals that cause tissue damage in frozen foods. Furthermore, the formation of large ice crystals can destroy the muscle fibers of frozen fish [20]. Conversely, the rapid rates of heat loss and ice production in the quick freezing method cause minimal disturbance to the cell walls [33].

According to the results of this study, quick freezing successfully inhibits changes in aroma, color, flavor, texture, and overall acceptance. Quick freezing can remove heat faster than slow freezing. The heat removal rate determines the crystal growth rate [34]. In addition, there is less disruption to the cell walls in the quick freezing system owing to the rapid rate of heat removal and ice formation [33]. Leygonie et al. [35], Li et al. [36], and Wang et al. [37] mentioned that quick freezing is a key technology for maintaining the quality of frozen food, whereas slow freezing has been known to result in the formation of large extracellular ice crystals, which may cause severe tissue damage in frozen foods [32]. Alizadeh et al. [20] reported that the use of a slow-freezing method for fish resulted in the development of large ice crystals and significantly destroyed the muscle fibers.

As the sensory evaluation results of quick freezing are better than slow freezing, particularly in texture, we used quick freezing as the freezing method for the test HMR products.

### 3.5. Nutritional Composition of Test HMR Product

The nutritional quality of the test HMR products was measured. Specifically, the proximate, amino acid, and fatty acid profiles of the test HMR were evaluated to determine the nutritional value of the test HMR product. The results of the proximate analysis revealed that the test HMR product contains both macro and micronutrients, including calcium, protein, fat, iron, and potassium (Table 6). Mouritsen and Styrbaek [38] reported that octopus contains macro and micronutrients such as protein, fat, calcium, iron, magnesium, potassium, sodium, and zinc. These nutrients can be a source of nutrition and contribute to daily dietary needs. Moreover, carbohydrates, proteins, and fats, which were all detected in the HMR test, act as a source of energy for specific health-maintaining functions. According to Murthy and Singh [39], macronutrients from food, including crude fat, carbohydrates, and proteins, are necessary for daily activities. These results show that the test HMR contains essential nutrients for humans and contributes to daily nutritional needs.

The amino acid profile results showed that non-essential amino acid contents were higher than the essential contents, in which the total amino acid contents were 14.08 g/100 g (Table 7). Compared to other seafood HMR products, total amino acids of test HMR products were higher. In the study of Tirtawijaya et al. [2], the total amino acid of seafood HMR products consisting of mackerel and radish was 12.94 g/100 g, while in the study of Negara et al. [5], the seafood HMR product consisting of a mixture of adductor muscle of the pen shell, common squid meat, and sauce contained 7.93 g/100 g total amino acids. The Glutamic acid was the predominant amino acid in the test HMR product (approximately 17.33%). Glutamic acid is prevalent in fishery products. Li et al. [40] and Li and Wu [41] reported that glutamine and glutamic acid are prominent amino acids. These amino acids can be used as a source of energy for the immune system [42]. Furthermore, leucine, arginine, and threonine were the dominant essential amino acids. The presence of both essential and non-essential amino acids in the test HMR product indicates that the product contained amino acids that are essential for humans.

These amino acids have been reported to be beneficial for human health, especially for improving the immune system and reducing the risk of cardiovascular disease [43]. Akram et al. [44] reported that amino acids can help repair tissue, support growth, and provide energy. Moreover, the presence of some amino acids can affect the taste of the product. Sarower et al. [45] reported that the presence of glycine, alanine, aspartic acid, and glutamic acid in foods gave them a sweet taste. Overall, the amino acid analysis revealed that the test HMR product contains essential amino acids and contributes to human daily amino acid requirements.

The fatty acid composition analysis showed that test HMR products contain saturated fatty acids (SFAs), polyunsaturated fatty acids (PUFAs), and monounsaturated fatty acids (MUFAs) (Table 8). Overall, the PUFA contents were the highest, followed by total SFAs and MUFAs. Of the PUFAs, total omega 3 content was higher than omega 6 content. Docosahexaenoic acid (DHA) was the most abundant fatty acid in the test HMR products, followed by palmitic acid and EPA. The results of fatty acids contents in this study agreed with study of Negara et al. [5] and Tirtawijaya et al. [2]. They found that the seafood HMR products contain omega 3, omega 6, DHA, and EPA. Both DHA and EPA contribute to human health. According to Swanson et al. [46], DHA and EPA are important for retinal, neuronal, and immune functions, and may also affect many aspects of cardiovascular function, including inflammation, peripheral artery disease, major coronary events, and anticoagulation. Moreover, DHA and EPA can prevent Alzheimer’s disease. Meanwhile, omega-6 fatty acids play a vital role in many physiological functions [47], including reducing the risk of heart disease, lowering total cholesterol levels, lowering “bad” cholesterol levels, raising “good” cholesterol levels, and reducing cancer risk. According to these results, the fatty acid composition demonstrated that the HMR product had high nutritional value, implying that it might contribute to daily fatty acid requirements.

### 3.6. Shelf-Life Estimation

As a food product, the shelf-life of the test HMR product was estimated using the Arrhenius approach. To estimate the shelf-life, we used three different storage temperatures: −13 °C, −18 °C, and −23 °C. For the shelf-life estimation, the overall acceptance, TBARS, TBC, *Salmonella* spp., and *S. aureus* contents were observed for 90 days. Before measuring these parameters, the test HMR product was reheated. All data were analyzed using a visual shelf-life simulator from the MFDS.

The results of overall acceptance at different storage temperatures showed a decreasing trend during storage, whereas TBARS and TBC increased during storage (Table 9). When stored at −13 °C, the overall acceptance of the test HMR started to decrease significantly (*p* < 0.05) on day 30, as compared with day 0. Conversely, at −18 °C and −23 °C, overall acceptance was not significantly (*p* < 0.05) different throughout the shelf-life estimation. The panelists gave the test HMR product scores ranging from 7.33 to 8.63, categorizing the product into “like moderately” to “like very much”, according to the hedonic scale. At −18 °C and −23 °C, the sensory characteristics of the test HMR product were better maintained at −13 °C during storage. While a decrease in overall acceptance was observed at −13 °C, the score of overall acceptance was categorized as moderate, indicating that the test HMR product still had good sensory characteristics.

The maintenance of sensory characteristics during storage might be the result of quick freezing. Quick freezing results in the formation of fine ice crystals within muscle cells, while slow freezing leads to the formation of ice crystals outside the muscle cells [20,35,48]. The formation of ice crystals outside of muscle cells distorts the structure of frozen products [35,49,50,51], which decreases sensory properties [52,53]. Therefore, by applying quick freezing, a decrease in sensory properties can be prevented.

The VBN and TBARS results showed an increase during the storage period. At −13 °C, a significant increase (*p* < 0.05) was observed on days 60 and 45 for VBN and TBARS, respectively. Meanwhile, at −18 °C and −23 °C, the TBARS increased significantly (*p* < 0.05) on day 75, whereas for the VBN, no significant (*p* < 0.05) difference was observed throughout the shelf-life estimation at these temperatures. The increases in VBN and TBARS are related to the oxidation of proteins and lipids, as well as the spoilage rate of the test HMR product. During storage, higher temperatures lead to faster oxidation processes, as compared with lower temperatures. While an increase was observed in the test HMR product during storage, the TBARS values were less than 3 mg MDA/kg, indicating that the test HMR products were still perfect products [27].

The microbial analysis showed different results at different temperatures. At −13 °C, the TBC significantly (*p* < 0.05) increased on day 15, while at −18 °C and −23 °C, the TBC increased significantly (*p* < 0.05) on days 60 and 90, respectively. Meanwhile, *Salmonella* spp. and *S. aureus* were not detected at any temperature during storage. These results indicate that lower temperatures could inhibit microbial activity. During the storage period, the number of TBCs was <5 log CFU/g, categorizing it as a satisfactory product [54].

The prediction of shelf life resulted in three model equations. For overall acceptance, the model equation was y = 60.17 − 17317.51x, while for VBN, TBARS and TBC, the model equations were y = 24.98 − 8142.78x, y = 8.06 − 3301.29x, and y = 41.41 − 12424.21x, respectively. The shelf-life simulation, according to overall acceptance, VBN, and TBC, resulted in 18.31 months. To predict the final shelf-life, a safety factor (0.8) was used to multiply the Arrhenius calculation result [55]. A safety factor is used to consider temperature changes during market operations. Overall, the shelf-life estimation for the test HMR product was 15 months. This indicates that the test HMR products will maintain good overall acceptance, VBN, and TBC for 15 months.

## 4. Conclusions

This study revealed that the application of steam and smoke processes to produce new HMR *jjukkumi bokkeum* resulted in a high-quality product. Specifically, steam and smoke processes successfully maintained the physicochemical properties of web-foot octopus as a raw material. During these processes, the freshness of the web-foot octopus was preserved, and the oxidation of proteins and lipids was avoided. These conditions affect the reducing rate of spoilage.

The application of steam and smoke successfully produced an HMR *jjukkumi bokkeum* with high nutritional value. This HMR product contains good fatty acids and amino acids that can contribute to daily nutritional requirements. The sensory characteristics of the test HMR product were maintained after reheating, and have a shelf-life of 15 months. During these months, the sensorial, physicochemical, and microbial properties of new HMR products are maintained at levels that are acceptable for consumption. The findings of this study can be applied to the seafood industry to produce new HMR products.

## Figures and Tables

**Table 1:** Ingredients for *jjukkumi bokkeum* sauce.

No.	Ingredients	(%)
1.	Corn syrup	8.22
2.	Fermented anchovy sauce	1.93
3.	Garlic powder	3.22
4.	Gochujang	3.22
5.	Korean beef stock	0.64
6.	Monosodium glutamate	0.32
7.	Oligosaccharide	4.83
8.	Pepper	0.06
9.	Purified water	45.12
10.	Red pepper powder	16.11
11.	Soy sauce	6.45
12.	Sugar	9.86

**Table 2:** The nine-point hedonic scale used to evaluate sensory characteristics of test HMR products.

Sensorial Characteristics	Hedonic Scale								
	1	2	3	4	5	6	7	8	9
Aroma	Rancid Extremely	Rancid Very Much	Aromatic Moderately	Aromatic Slightly	Musty	Savoury Moderately	Savoury Slightly	Mild Very Much	Mild Extremely
Color	Dislike Extremely	Dislike Very Much	Dislike Moderately	Dislike Slightly	Neither Like nor Dislike	Like Slightly	Like Moderately	Like Very Much	Like Extremely
Flavor	Uncooked	Uncooked Slightly	Brunt Moderately	Brunt Slightly	Fatty	Salty Moderately	Salty Slightly	Tasty Very Much	Tasty Extremely
Texture	Hard Extremely	Hard Moderately	Dry Moderately	Dry Slightly	Dry	Soft Slightly	Soft	Chewy Moderately	Chewy
Overall acceptance	Dislike Extremely	Dislike Very Much	Dislike Moderately	Dislike Slightly	Neither Like nor Dislike	Like Slightly	Like Moderately	Like Very Much	Like Extremely

**Table 3:** Response surface model equations of web-foot octopus for steam and smoke processes.

Treatment	Quadratic Polynomial Model Equations	*R* ^2^
Steam	−421.7 + 8.927X_1_ + 5.77X_2_ − 0.04668X_1_^2^ − 0.7921X_2_^2^ − 0.0250X_1_X_2_	98
Smoke	−59.93 + 1.810X_1_ + 0.951X_2_ − 0.012708X_1_^2^ − 0.03217X_2_^2^ − 0.00333X_1_X_2_	97

**Table 4:** Effect of steam and smoke processes on the physicochemical characteristics of web-foot octopus.

Parameters	Raw Octopus	Steamed-Smoked Octopus
pH	7.24 ± 0.1	7.38 ± 0.03
VBN (mg%)	6.32 ± 0.01	3.69 ± 0.25
TBARS (mg MDA/kg)	1.10 ± 0.03	1.04 ± 0.01

Note: Volatile basic nitrogen (VBN); Thiobarbituric acid reactive substances (TBARS). Data are presented as mean ± standard deviation.

**Table 5:** Sensory characteristics of the test seafood home meal replacement product before freezing, and after quick freezing and slow freezing.

Sensorial Characteristic	Fresh Product	Quick Freezing	Slow Freezing
Aroma	8.79 ± 0.05 ^a^	8.76 ± 0.03 ^a^	8.74 ± 0.02 ^a^
Color	8.66 ± 0.10 ^a^	8.64 ± 0.04 ^a^	8.62 ± 0.09 ^a^
Flavor	8.74 ± 0.05 ^a^	8.71 ± 0.03 ^a^	8.69 ± 0.05 ^a^
Texture	8.72 ± 0.04 ^a^	8.70 ± 0.06 ^a^	8.31 ± 0.10 ^b^
Overall acceptance	8.63 ± 0.25 ^a^	8.62 ± 0.06 ^a^	8.61 ± 0.07 ^a^

Data are presented as mean ± standard deviation. The means of each sensory property denoted with different letters are significantly different according to Duncan’s test (*p* < 0.05).

**Table 6:** Nutritional composition of the home meal replacement product containing web-foot octopus with sauce.

Parameters	Unit (per 100 g)	Contain
Calcium	mg	18
Calories	kcal	151.6
Carbohydrate	g	23.7
Cholesterol	g	0.6
Crude fat	g	9.7
Crude protein	g	13.5
Dietary fiber	g	0.8
Iron	mg	0.7
Trans fat	g	2.0
Potassium	g	0.3
Saturated fat	g	0.4
Sodium	mg	8.7
Sugars	g	13.2
Trans fat	g	-
Vitamin D	g	-

Note: Symbol (-) means parameters were not detected.

**Table 7:** Amino acid profiles of the home meal replacement product containing web-foot octopus with sauce.

Amino Acids	g/100 g	%
Alanine	0.98	6.96
Aspartic acid	1.59	11.29
Cysteine	0.16	1.14
Glutamic acid	2.44	17.33
Glycine	1.20	8.52
Proline	0.73	5.18
Serine	0.69	4.90
Tyrosine	0.39	2.77
Total Non-Essential	8.18	58
Arginine	0.85	6.04
Histidine	0.29	2.06
Isoleucine	0.69	4.90
Leucine	1.26	8.95
Lysine	0.48	3.41
Methionine	0.27	1.92
Phenylalanine	0.57	4.05
Threonine	0.72	5.11
Tryptophan	0.14	0.99
Valine	0.63	4.47
Total Essential	5.9	42
Total Amino Acid	14.08	100

**Table 8:** Fatty acid profiles of home meal replacement product containing web-foot octopus with sauce.

Fatty Acids	%
Capric acid	0.01
Lauric acid	0.01
Myristic acid	3.18
Pentadecanoic acid	0.30
Palmitic acid	22.12
Magaric acid	0.10
Stearic acid	1.68
Arachidic acid	0.04
Heneicosylic acid	0.02
Lignoceric acid	1.67
Myristoleic acid	0.06
Pentadecenoic acid	0.05
Palmitoleic acid	5.33
Magaoleic acid	0.15
Oleic acid	16.54
Linoleic acid	0.28
γ-Linolenic acid	0.07
Linolenic acid	0.28
Eicosenoic acid	2.05
Eicosadienoic acid	0.20
Dihomoδ-Linoleicacid	0.06
Eicosatrienoic acid	0.06
Arachidonic acid	0.71
Erucic acid	0.34
DHA	23.60
EPA	20.40
ƩSFA	29.13
ƩPUFA	46.15
ƩMUFA	24.72
Ʃ ω3	44.34
Ʃ ω6	1.81

Notes: ƩSFA, saturated fatty acid; ƩPUFA, polyunsaturated fatty acid; ƩMUFA, monounsaturated fatty acid; DHA, docosahexaenoic acid; EPA, eicosapentaenoic acid.

**Table 9:** Sensorial, chemical, and biological characteristics of the home meal replacement product containing web-foot octopus with sauce during storage at three different temperatures for 90 days.

Temperature (°C)	Day	Overall Acceptance	VBN (mg%)	TBARS (mg MDA/kg)	TBC (log CFU/g)	*Salmonella* spp.	*S. aureus*
−13	0	8.63 ± 0.25 ^a^	3.70 ± 0.09 ^a^	1.06 ± 0.03 ^a^	4.06 ± 0.10 ^a^	-	-
	15	8.44 ± 0.03 ^ab^	3.79 ± 0.17 ^ab^	1.27 ± 0.03 ^ab^	4.19 ± 0.01 ^b^	-	-
	30	8.21 ± 0.02 ^bc^	3.84 ± 0.16 ^ab^	1.45 ± 0.05 ^ab^	4.24 ± 0.02 ^b^	-	-
	45	8.00 ± 0.05 ^c^	3.96 ± 0.21 ^ab^	1.64 ± 0.04 ^bc^	4.48 ± 0.02 ^c^	-	-
	60	7.60 ± 0.22 ^d^	4.09 ± 0.27 ^ab^	1.97 ± 0.04 ^cd^	4.60 ± 0.02 ^d^	-	-
	75	7.47 ± 0.25 ^d^	4.21 ± 0.23 ^b^	2.22 ± 0.02 ^de^	4.75 ± 0.02 ^e^	-	-
	90	7.33 ± 0.12 ^d^	4.39 ± 0.25 ^b^	2.57 ± 0.05 ^e^	4.88 ± 0.09 ^f^	-	-
−18	0	8.63 ± 0.25 ^a^	3.70 ± 0.09 ^a^	1.06 ± 0.03 ^a^	4.06 ± 0.10 ^a^	-	-
	15	8.61 ± 0.02 ^a^	3.76 ± 0.01 ^a^	1.25 ± 0.02 ^a^	4.09 ± 0.01 ^a^	-	-
	30	8.56 ± 0.03 ^a^	3.79 ± 0.15 ^a^	1.39 ± 0.03 ^ab^	4.12 ± 0.02 ^a^	-	-
	45	8.53 ± 0.03 ^a^	3.85 ± 0.23 ^a^	1.46 ± 0.02 ^ab^	4.13 ± 0.02 ^a^	-	-
	60	8.49 ± 0.01 ^a^	3.90 ± 0.24 ^a^	1.49 ± 0.05 ^ab^	4.18 ± 0.02 ^b^	-	-
	75	8.45 ± 0.02 ^a^	3.95 ± 0.24 ^a^	1.85 ± 0.04 ^bc^	4.20 ± 0.01 ^c^	-	-
	90	8.40 ± 0.16 ^a^	3.99 ± 0.23 ^a^	2.26 ± 0.06 ^c^	4.22 ± 0.02 ^d^	-	-
−23	0	8.63 ± 0.25 ^a^	3.70 ± 0.09 ^a^	1.06 ± 0.03 ^a^	4.06 ± 0.10 ^a^	-	-
	15	8.61 ± 0.04 ^a^	3.74 ± 0.02 ^a^	1.15 ± 0.02 ^a^	4.08 ± 0.02 ^ab^	-	-
	30	8.59 ± 0.04 ^a^	3.77 ± 0.16 ^a^	1.20 ± 0.09 ^a^	4.09 ± 0.01 ^ab^	-	-
	45	8.57 ± 0.03 ^a^	3.81 ± 0.18 ^a^	1.25 ± 0.02 ^ab^	4.12 ± 0.02 ^ab^	-	-
	60	8.55 ± 0.03 ^a^	3.84 ± 0.18 ^a^	1.35 ± 0.04 ^ab^	4.13 ± 0.01 ^ab^	-	-
	75	8.53 ± 0.02 ^a^	3.86 ± 0.18 ^a^	1.55 ± 0.02 ^b^	4.15 ± 0.01 ^ab^	-	-
	90	8.50 ± 0.16 ^a^	3.88 ± 0.19 ^a^	1.90 ± 0.12 ^c^	4.17 ± 0.01 ^b^	-	-

Data are presented as mean ± standard deviation. Means in each column at each temperature group marked with different letters (a–f) differed significantly according to Duncan’s test (*p* < 0.05).

## Data Availability

Data supporting reported results are available upon request.
